# Antiferromagnetic correlations in the metallic strongly correlated transition metal oxide LaNiO_3_

**DOI:** 10.1038/s41467-017-02524-x

**Published:** 2018-01-03

**Authors:** H. Guo, Z. W. Li, L. Zhao, Z. Hu, C. F. Chang, C.-Y. Kuo, W. Schmidt, A. Piovano, T. W. Pi, O. Sobolev, D. I. Khomskii, L. H. Tjeng, A. C. Komarek

**Affiliations:** 1 Max-Planck-Institute for Chemical Physics of Solids, Nöthnitzer Street 40, 01187 Dresden, Germany; 2 Forschungszentrum Jülich GmbH, Jülich Centre for Neutron Science at ILL, CS 20156, 71 avenue de Martyrs, 38042 Grenoble, France; 3 Institut Laue-Langevin (ILL), 71 avenue des Martyrs, 38042 Grenoble, France; 4 National Synchrotron Radiation Research Center (NSRRC), 101 Hsin-Ann Road, 30077 Hsinchu, Taiwan; 5 Georg-August-Universität Göttingen, Institut für Physikalische Chemie, Tammannstrasse 6, 37077 Gottingen, Germany; 6 Physics Institute II, University of Cologne, Zülpicher Street 77, 50937 Cologne, Germany

## Abstract

The material class of rare earth nickelates with high Ni^3+^ oxidation state is generating continued interest due to the occurrence of a metal-insulator transition with charge order and the appearance of non-collinear magnetic phases within this insulating regime. The recent theoretical prediction for superconductivity in LaNiO_3_ thin films has also triggered intensive research efforts. LaNiO_3_ seems to be the only rare earth nickelate that stays metallic and paramagnetic down to lowest temperatures. So far, centimeter-sized impurity-free single crystal growth has not been reported for the rare earth nickelates material class since elevated oxygen pressures are required for their synthesis. Here, we report on the successful growth of centimeter-sized LaNiO_3_ single crystals by the floating zone technique at oxygen pressures of up to 150 bar. Our crystals are essentially free from Ni^2+^ impurities and exhibit metallic properties together with an unexpected but clear antiferromagnetic transition.

## Abstract (web summary)

The phase transitions of rare earth nickelates have attracted intensive study as they arise from the complex interplay of charge, spin and lattice degrees of freedom. Here Guo et al. present evidence that LaNiO_3_ has an unanticipated magnetically ordered metallic phase.

## Introduction

The rare earth nickelates *R*NiO_3_ (*R* = rare earth, Y) with the high Ni^3+^ oxidation state have continued to attract enormous interest due to the famous bandwidth controlled metal-insulator (MI) transition and associated unusual charge and spin-order phenomena occurring in this system^1,2^ with even the possibility for multiferroicity^3^. The description of the underlying physics of these phenomena turned out to be a true intellectual challenge and it is not a surprise that important theoretical concepts have been (and still need to be) developed along the way^4–12^. More recently, the prediction of high-*T*_c_ superconductivity in LaNiO_3_-based heterostructures^13^ has triggered a flurry of new activities on LaNiO_3_–La*M*O_3_ superlattices (*M* = other metal ion)^14,15^.

Apart from LaNiO_3_, all *R*NiO_3_ (*R* = Pr-Lu, Y) compounds exhibit a MI transition at lower temperatures^1,2^ with an insulating antiferromagnetic ground state. With decreasing *R*-ionic radius, the octahedral tilts become larger. Thus, the Ni–O–Ni bond angles become smaller, which alters the electronic bandwidth and the magnetic exchange interactions. Whereas an enhancement of the insulating properties and an increase of the MI transition temperature *T*_MI_ can be observed for decreasing *R*-ionic radius, the antiferromagnetic transition temperature and the strength of the exchange interactions decrease with decreasing *R*-ionic radius and Ni–O–Ni bond angles. With La having the largest ionic radius of the series, one might expect the strongest antiferromagnetic properties for LaNiO_3_. However, so far it was reported that LaNiO_3_ does not show any magnetic order, and thus, violates the trend. It remains a paramagnetic metal down to lowest temperatures with an enhanced effective mass^1,2,16,17^.

Here, we report on the successful growth of large LaNiO_3_ single crystals by the floating zone technique. Electrical resistivity and Hall effect measurements on our single crystals show that LaNiO_3_ is intrinsically not a bad metal as recently discussed for the *R*NiO_3_ (*R* = Pr-Lu, Y) compounds in their paramagnetic phase^10^. On the contrary, we found that LaNiO_3_ has a high conductivity. Moreover, we were able to observe bulk antiferromagnetism in magnetization, specific heat, and neutron scattering experiments. Thus, LaNiO_3_ appears to be a highly metallic and antiferromagnetic transition metal oxide—a rather rare combination in oxides. Special about LaNiO_3_ is that it is also very close to an insulating state, making LaNiO_3_ an intriguing quantum material, probably close to a quantum critical point, where strong local electronic correlations at the Ni sites are likely to interfere in an intricate manner with Fermi surface effects.

## Results

### Crystal growth and characterization

**Fig. 1:**
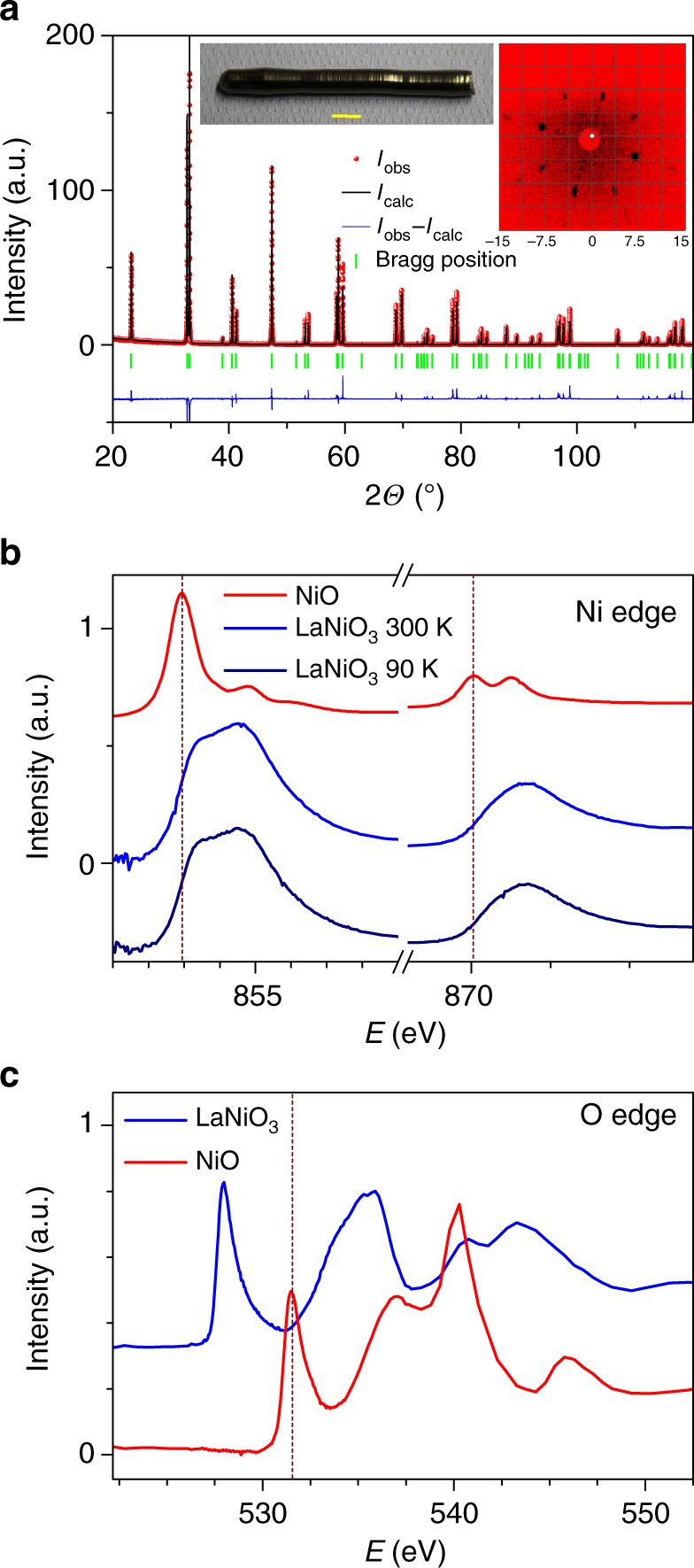
Characterization of LaNiO_3_. **a** Powder X-ray diffraction pattern of a powdered LaNiO_3_ single crystal. The inset shows a photo of the LaNiO_3_ single crystal and its Laue diffraction pattern. A length scale of 1 cm is indicated by the yellow bar within the photo of the crystal and the distance between two lines in the Laue pattern amounts to 3.75 cm. **b** The Ni-L_2,3_ XAS spectra of the LaNiO_3_ single crystal measured at 90 and 300 K together with the spectrum of a NiO reference compound. **c** The O-K XAS spectra of our LaNiO_3_ single crystal measured at 300 K (blue line) together with the spectra of the NiO reference compound (red line)

Using high oxygen pressures of 130–150 bar, we were able to grow LaNiO_3_ single crystals with the floating zone technique, see inset of Fig. 1a. Further details can be found in the Methods section and in Supplementary Notes 1 and 2, in Supplementary Figures 1 and 2, and in Supplementary Movie 1. Powdered crystals exhibit no impurity phases within the accuracy of powder X-ray diffraction measurements, see Fig. 1a. The single crystallinity of our LaNiO_3_ crystal is confirmed by a series of Laue diffraction patterns from different directions and across the length of this crystal as well as by single crystal X-ray diffraction, see inset of Fig. 1a and Supplementary Figure 1, as well as Supplementary Note 3, Supplementary Figure 3, and Supplementary Table 1. Due to the occurrence of a cubic to rhombohedral phase transition (*Pm* $\overline 3$ *m* $\to$ *R* $\overline 3$ *c*) somewhat below 1100 K^2^, our floating zone grown single crystals are twinned.

Figure 1b shows the Ni-L_2,3_ X-ray absorption spectroscopy (XAS) data of the LaNiO_3_ single crystal taken at 300 and 90 K together with that of a NiO single crystal serving as a Ni^2+^ reference compound. We have removed the La-M_4_ white lines located at 850.6 eV from the LaNiO_3_ spectra using the La-M_4_ spectrum of LaCoO_3_. It is well known that XAS spectra at the L_2,3_ edge of transition metal oxides are highly sensitive to the valence state, in particular, an increase of the valence state of the transition metal ion by one causes a shift of the XAS L2_2,3_ spectra by one or more eV toward higher energies^18^. The more than one eV higher energy shift between the spectra of NiO and LaNiO_3_ indicates the formal Ni^3+^ valence state in LaNiO_3_^18,19^, with the note that the spectrum cannot be interpreted in terms of a Ni 3d^7^ configuration, but, rather by a coherent mixture of 3d^8^ and 3d^8^ $\underline L$ $\underline L$ configurations^4,5,9,12^, where each $\underline L$ denotes a hole in the oxygen ligand. Here we can exclude Ni^2+^ impurities in our LaNiO_3_ single crystal—otherwise the sharp main peak of the Ni^2+^ impurity spectrum would have been visible as a sharp shoulder at the leading edge^18^. At the O-K edge, the pre-edge peak is shifted by about 1 eV to higher energies when going from Ni^3+^ to Ni^2+^ ^18,19^. Figure 1c shows the O-K XAS spectra of our LaNiO_3_ single crystal (blue) and the NiO reference compound (red). The O-K XAS spectra demonstrate even more clearly that there is no spectral feature from Ni^2+^ impurities and thus that our as-grown LaNiO_3_ single crystals are highly stoichiometric. This is further confirmed by thermogravimetric and inductively coupled plasma optical emission spectroscopy (ICP-OES) measurements, see Methods and Supplementary Note 2.

### Temperature dependence of physical properties

**Fig. 2:**
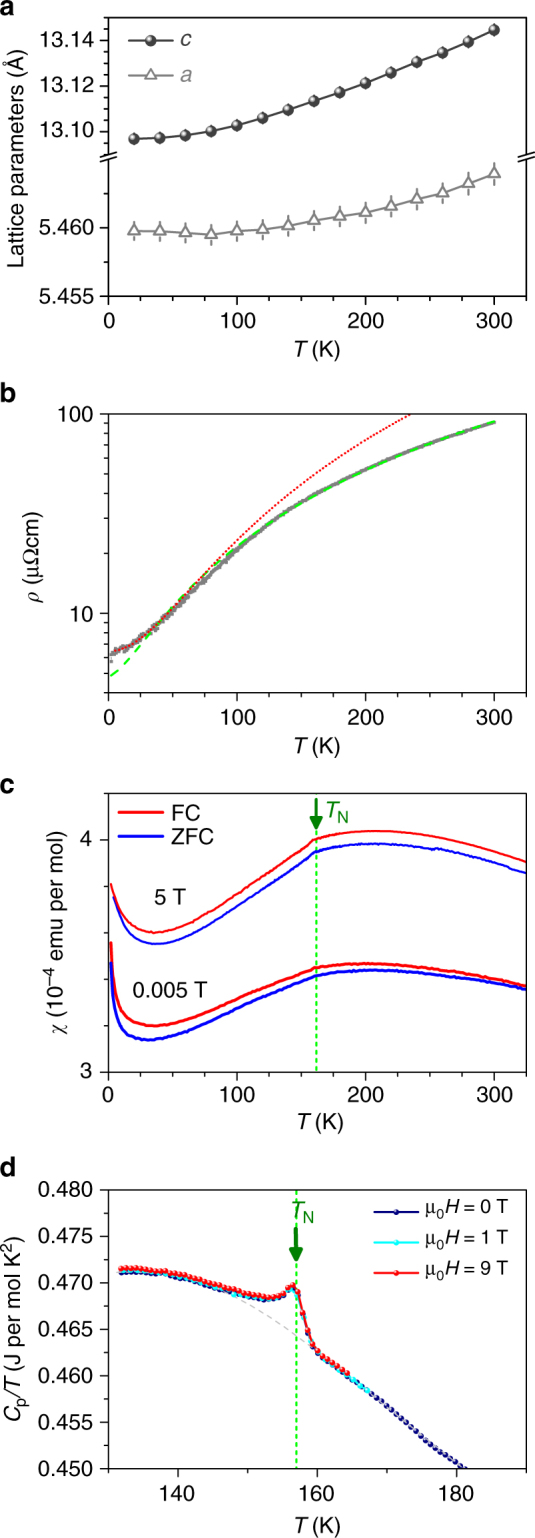
Temperature dependence of physical properties of LaNiO_3_. **a** Lattice parameters of our LaNiO_3_ as a function of temperature (space group *R* $\bar 3$ *c*). The error bars are the standard deviations obtained from Rietveld refinement using the Fullprof software. **b** Electrical resistivity of LaNiO_3_ (gray). The red line indicates a fit to $\rho _0 + AT^n$ (for *T <*35 K) and the dashed green line is a fit over the entire temperature range. **c** Magnetic susceptibility of LaNiO_3_ under field cooled (FC) and zero-field cooled (ZFC) conditions for applied fields of μ_0_ *H* = 0.005 T and 5 T. The high- and low-field measurements both together indicate an antiferromagentic transition at *T*_N_ that is an intrinsic property of LaNiO_3_. **d** Specific heat measurements of LaNiO_3_ in fields of 0 T, 1 T, and 9 T. These measurements further corroborate that the antiferromagnetic transition is a bulk property of LaNiO_3_

In Fig. 2a, we show the temperature dependence of the lattice parameters of our LaNiO_3_ single crystal that has been powdered and measured by means of powder X-ray diffraction. There is no clear indication for the presence of a structural anomaly which otherwise occurs readily in the other nickelates *R*NiO_3_ with smaller rare earths (*R* = Pr-Lu, Y) when cooling through the MI transition^1,2^.

The electrical resistivity *ρ* of our LaNiO_3_ single crystal that is shown in Fig. 2b is in the μΩ cm range, reaching ~6 μΩ cm at low temperatures, i.e., distinctly lower than that of LaNiO_3_ powder samples^1,2,16,17^ or LaNiO_3_ thin films^14^ or of crystals grown under 50 bar oxygen pressure^20^. Thus, our LaNiO_3_ single crystals are more metallic, see also Supplementary Note 4 and Supplementary Figure 4(a). Also the measurement of the Hall effect on the single crystal LaNiO_3_ (for temperatures between 2 and 300 K) yields high carrier densities of about 4.8 × 10^28^ m^−3^, corresponding to ~2.7 (close to 3) electrons per formula unit, consistent with the 3+ valence of the Ni ions in LaNiO_3_. Up to ~35 K, the low-temperature behavior of the resistivity is Fermi-liquid-like, *ρ*(*T*) = $\rho _0 + AT^n$, with $\rho _0$ ~6.45 μΩ cm, *A* = 1.62.10^−3^ μΩ cm K^−2^, and *n* ~2.0, see Fig. 2b. The value of *A* is of the same order as reported in literature^21^. A fit over the entire temperature range gives an exponent *n* ~1.50(1) with the residual resistivity $\rho _0$ ~4.83 μΩ cm, similar to the value of the fit at low temperatures. Most probably this behavior is a signature that LaNiO_3_ is close to a quantum critical point (cf. the results for PrNiO_3_ under pressure^21^).

Figure 2c displays the temperature dependence of the magnetic susceptibility *χ* of our LaNiO_3_ single crystal, see also Supplementary Note 4 and Supplementary Figure 4(b). First of all, we notice that it shows a significantly smaller low-temperature upturn than reported previously for powder and ceramic samples^16,17^, which confirms unprecedented high quality of our single crystals. Surprisingly, *χ* exhibits an anomalous kink at ~157 K, which we take as an indication for a hitherto unknown antiferromagnetic transition in LaNiO_3_. That this anomaly is not simply caused by a signal from a tiny fraction of a magnetic impurity phase (which is so small that it is not visible in our powder X-ray diffraction measurements) can be excluded by our specific heat (*C*_p_) measurements. As can be seen in Fig. 2d, there is a small but clearly visible anomalous peak at ~157 K in $C_{\mathrm{p}}/T$. Also the resistivity data excludes that the anomaly in the susceptibility is caused by the presence of an oxygen-deficient LaNiO_3_ minority phase that becomes antiferromagnetic and insulating at low temperatures^22,23^: we do not observe an upturn or a slowing down of the decrease in the resistivity on cooling. Moreover, our samples have resistivities in the μΩ cm range and have conductivities higher than reported so far. All these support the notion that the transition is an intrinsic bulk property and not due to an impurity phase. The low-temperature behavior of the specific heat supports a Fermi-liquid type of the ground state of LaNiO_3_, *C*_p_(*T*) = $\gamma T + \beta T^3$, with $\gamma$ ~17 mJ mol^−1^ K^−2^, consistent with the value 18 mJ mol^−1^ K^−2^ reported before^17^, and with *β* = 1.87(5) × 10^−4^ J mol^−1^ K^−4^, thus yielding a Debye temperature ${\theta _{\mathrm{D} }= (12\pi ^4NR/5\beta )^{1/3}}$ = 373 K, where *N* is the number of atoms in the chemical formula and *R* is the ideal gas constant.

### Neutron scattering experiments

**Fig. 3:**
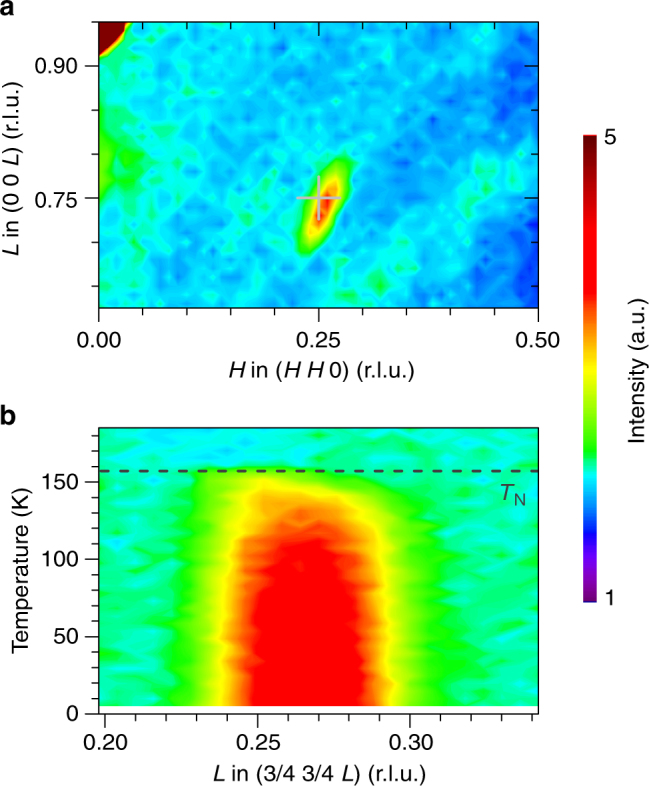
Single crystal neutron-diffraction measurements. **a** Neutron scattering intensities within the (*H H L*) plane of reciprocal space (in pseudocubic notation) of the LaNiO_3_ single crystal. These intensites were obtained at 1.6 K. **b** The temperature dependence of a quarter-integer peak as measured in (3/4 3/4 *L*) scans for different temperatures. The dashed line indicates the transition temperature (*T*_N_) observed in the specific heat

The availability of sizeable LaNiO_3_ single crystals also enabled us to study this intriguing system by means of neutron diffraction and inelastic neutron scattering. These experiments were performed at the Thales, IN12 and IN8 spectrometers at the ILL in Grenoble, France. Within elastic scans, we were able to observe quarter-integer peaks at low temperatures, see Fig. 3a. These quarter-integer peaks (in pseudocubic notation) resemble those found in powder neutron-diffraction experiments within the insulating regime of *R*NiO_3_^24^. The propagation vector observed for the insulating antiferromagnetic regime of *R*NiO_3_ amounts to (1/4 1/4 1/4) in pseudocubic notation (or (1/2 0 1/2) in orthorhombic notation)^2^. Moreover, the study of the temperature dependence of these quarter-integer peaks indicates an onset temperature that coincides with the magnetic ordering temperature *T*_N_ that we observed in susceptibility and specific heat measurements of LaNiO_3_, see Fig. 3b.

**Fig. 4:**
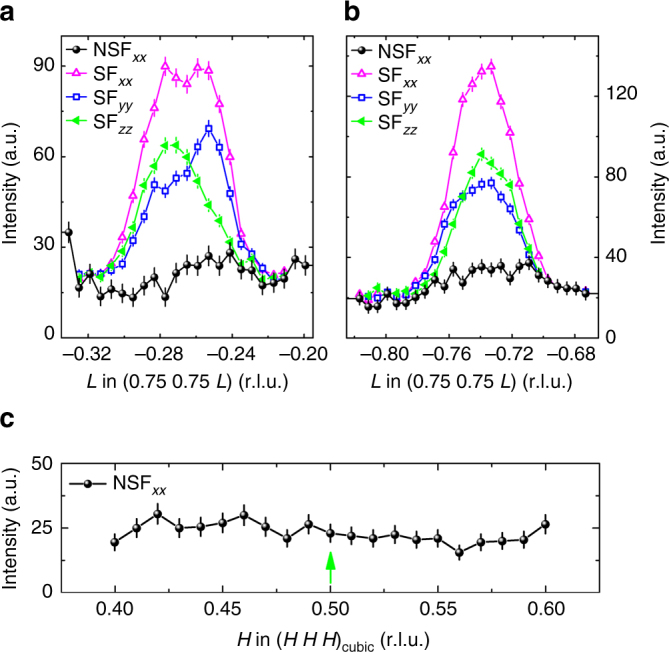
Polarized neutron-diffraction measurements. **a**, **b** Polarized neutron scattering experiments for the LaNiO_3_ single crystal at 2 K. Spin-flip (SF) and non-spin-flip (NSF) channels were measured for neutron spins parallel to the scattering vector (*x*-direction), perpendicular to the scattering plane (*z*-direction) or perpendicular to *x* and *z* (*y*-direction). **c** A structural scan at 2 K showing no indications for any charge ordering peak at the (0.5 0.5 0.5) position in pseudocubic notation (indicated by the green arrow). The intensity error bars are statistical error bars calculated by the square root of intensity

The magnetic origin of these quarter-integer peaks in LaNiO_3_ could be also confirmed by polarized neutrons at the IN12 spectrometer. In Fig. 4a & b *L*-scans across two quarter-integer peaks are shown for the three spin-flip (SF) channels and for a non-spin-flip channel. Only in the SF channels, neutron scattering intensities $\sigma _{x_ix_i}$ can be detected. This unambiguously shows the magnetic nature of these quarter-integer reflections in LaNiO_3_. Although we cannot detect a symmetry lowering from our high-resolution powder X-ray diffraction measurements—see Fig. 2a—the pseudocubic propagation vector for rhombohedral LaNiO_3_ is the same as the pseudocubic propagation vector for the orthorhombic insulating nickelates *R*NiO_3_ (*R* = Pr-Lu, Y). Based on the magnetic symmetry analysis for the high symmetry cubic structure with space group *Pm* $\overline 3$ *m* and for the propagation vector (1/4 1/4 1/4), a helical magnetic structure with moments spiraling perpendicular to the propagation vector is consistent with our single crystal neutron data, see Supplementary Note 2, Supplementary Tables 2 and 3, and Supplementary Figure 6. The size of the ordered moment amounts to ~0.3_B_, which indicates that magnetism in LaNiO_3_ is a bulk property and does not originate from a tiny (insulating) impurity phase. This small magnetic moment might explain why paramagnetic properties have been reported for LaNiO_3_ in the past^1,2,16,17^.

**Fig. 5:**
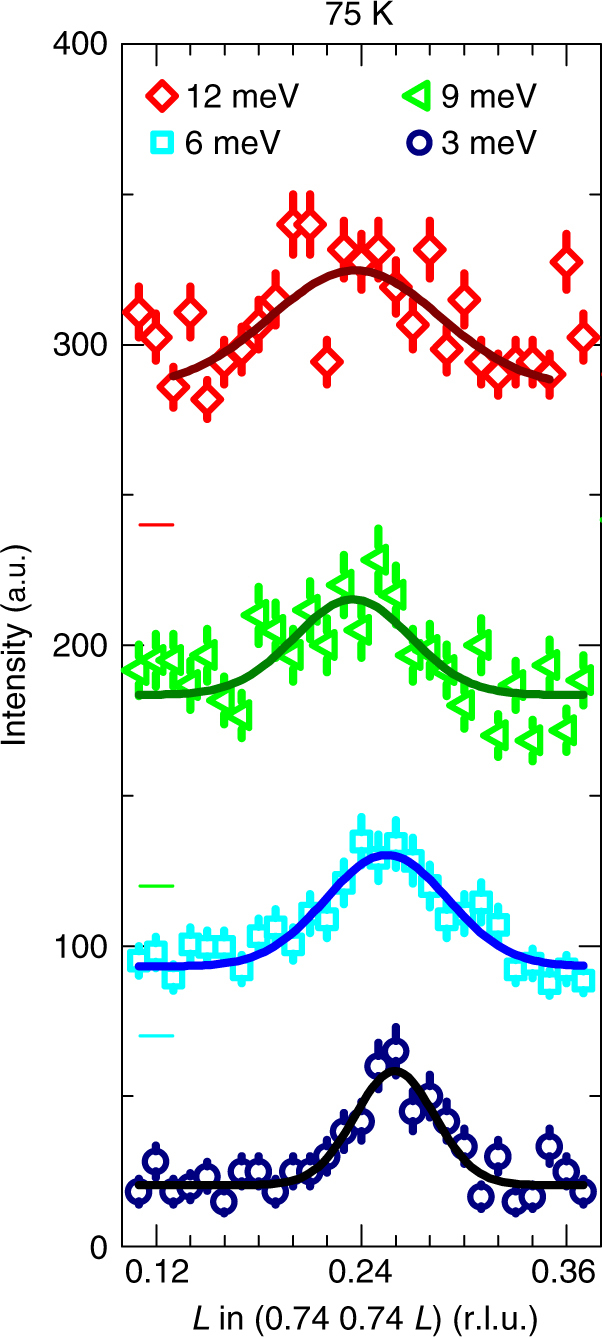
Magnetic excitations in LaNiO_3_. Inelastic neutron scattering intensities measured at 75 K for 3, 6, 9, and 12 meV energy transfer. The horizontal bars indicate the shift of the data in vertical direction. Up to 12 meV, the magnetic peaks become broader and damped, which is indicative for the appearance of fluctuations in this itinerant antiferromagnetic system. The intensity error bars are statistical error bars calculated by the square root of intensity

We also have been able to study and observe the magnetic excitations in LaNiO_3_ by means of inelastic neutron scattering, thereby also providing further support that the antiferromagnetism is a bulk property. As can be seen in Fig. 5, magnetic excitations are clearly visible up to at least 12 meV. With increasing energy transfer, the magnetic peaks become somewhat broader and damped, which is indicative for the appearance of fluctuations in this itinerant antiferromagnetic system. These fluctuations could be also responsible for a reduced ordered moment of ~0.3_B_ in LaNiO_3_.

## Discussion

LaNiO_3_ appears to be a rare case of an antiferromagnetic and metallic transition metal oxide with a fully three-dimensional (3D) crystal and electronic structure. Other systems like (La,Sr)_3_Mn_2_O_7_ and Ca_3_Ru_2_O_7_ have a lower electronic and structural dimensionality where the antiferromagnetic order is resulting from a stacking of ferromagnetic layers^25,26^. So far, the chromate system *A*CrO_3_ (*A* = Ca, Sr) and very recently, RuO_2_ are known to be the only other intrinsically antiferromagnetic and metallic transition metal oxides with such a fully 3D crystal and electronic structure^27,28^. The systems CaCrO_3_ and LaNiO_3_ have in common that the oxidation state of the transition metal ion is very high. Thus, the oxygen 2*p* to transition metal 3*d* charge-transfer energy here is apparently negative^5,12,29^ resulting in extreme 2*p*-3*d* covalency where the presence of holes in the oxygen band can effectively prevent the opening of the conductivity gap and at the same time mediate strongly the magnetic exchange interactions. However, in contrast to CaCrO_3_^27^, the rare earth nickelate LaNiO_3_ in single crystalline form is much more metallic and shows conductivities in the μΩ cm range. This is probably also true for RuO_2_^28^. Unique for LaNiO_3_ is that it is close to the insulating phase of *R*NiO_3_ (*R* = Pr-Lu, Y) indicating the importance of strong correlation effects, making it rather exceptional among all transition metal oxides.

**Fig. 6:**
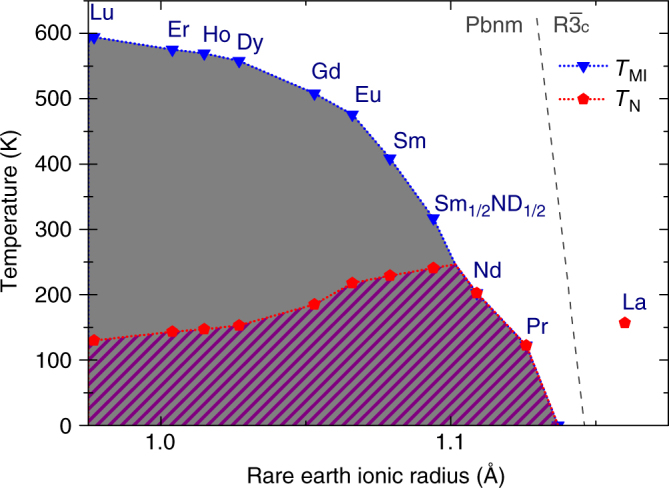
Tentative *R*NiO_3_ phase diagram. The blue triangles indicate the metal-insulator transition temperatures *T*_MI_ associated with the onset of charge ordering and the appearance of monoclinic distortions. The red symbols indicate the Néel temperatures *T*_N_ associated with the onset of antiferromagnetic ordering. Data points for other *R* than La were taken from literature^24,32^. The gray dashed curve subdivides orthorhombic (monclinic) and rhombohedral regimes

Although charge order, which plays a very important role in nickelates *R*NiO_3_ with small rare earth ions *R*, cannot be observed in our present X-ray diffraction (XRD) and neutron-diffraction measurements—see Fig. 4c—the presumably associated symmetry lowering of the structure that goes along with the antiferromagnetic order may produce only very weak new peaks in diffraction experiments. Some hints for this may in fact be found in a recent pair distribution function study using neutron diffraction^30^. Nevertheless, with the charge order effects being so weak, one could infer that LaNiO_3_ is perhaps better described using Fermi surface arguments^7,28^, while the other insulating nickelates *R*NiO_3_ with the smaller *R*-ionic sizes and with higher charge ordering temperatures can be more intuitively understood in terms of local charge or bond disproportionations^5,6,8,9,11,12^. Note, that recent ab initio calculations^31^ reproduced metallic and antiferromagnetic state of LaNiO_3_, and also predicted charge (or rather bond) disproportionation, whose magnitude is however below our experimental detection limit. One needs special dedicated experiments to probe for the eventual symmetry lowering with the appearance of inequivalent Ni ions. However, our main conclusion—the existence of antiferromagnetic ordering in highly metallic single crystals of LaNiO_3_, is confirmed by these calculations. According to our findings, we now also present a tentative *R*NiO_3_ phase diagram in Fig. 6.

## Methods

### Chemical synthesis

The LaNiO_3_ single crystal was grown under high oxygen pressures of 130–150 bar with a growth speed of 6–7 mm per h in a mirror furnace from Scidre that was equipped with a 5000 W Xe lamp and with counter-rotation of feeding and seeding rods—see Supplementary Movie 1. The seeding and feeding rods were synthesized by pressing appropriate mixtures of La_2_O_3_ and NiO into rods with roughly 10 cm length and 6 mm diameter. These rods were sintered at 800–1000 °C for several days. The temperature of the melting zone was measured in situ by means of a pyrometer and is close to 1800 °C at ~140 bar ${\rm {p}_O{_{_2}}}$.

### X-ray diffraction

Laue diffraction measurements were performed on a multiwire real-time back-reflection Laue camera from Multiwire Laboratories; see also Supplementary Note 1 and Supplementary Figure 1.

Powder X-ray diffraction measurements were performed on a Bruker D8 Discover A25 diffractometer, which is equipped with a Johansson monochromator for Cu K_α1_ radiation. A closed cycle helium cryostat (Phenix of Oxford Cryosystems) was used for temperature-dependent measurements.

Single crystal X-ray diffraction measurements have been performed on a twined single crystal of LaNiO_3_ using a Bruker D8 VENTURE single crystal X-ray diffractometer equipped with a bent graphite monochromator for Mo *K*_*α*_ radiation (about 3× intensity enhancement) and a Photon CMOS large area detector. A crystal with roughly 20 μm diameter has been measured and a multi-scan absorption correction has been applied to the data (minimum and maximum transmission: 0.6184 and 0.7519, respectively). A total of 12,328 (observed) reflections (*H*: −12→13, *K*: −13→9 and *L*: −32→31) have been collected with an internal *R*-value of 6.70%, a redundancy of 36.8, and with 98.85% coverage up to $2\Theta _{{\mathrm{max}}}$ = 123.6°. For the refinement, the Jana2006 program package was used. The goodness of fit of our crystal structure refinement amounts to 1.93, and the *R*- and weighted *R*-values amount to 2.29% and 5.79%, respectively. The refinement of the Ni and La occupancies yields an almost stoichiometric composition: La_0.995(12)_Ni_1.000(14)_O_3_. The structural parameters are listed in Supplementary Table 1 and the crystal structure is visualized in Supplementary Figure 3.

### Composition determined by ICP and TG measurements

Inductively coupled plasma optical emission spectroscopy measurements yields the following composition of LaNiO_3_: La_1.001(11)_Ni_0.999(4)_O_3+*δ*_. Moreover, thermogravimetric measurements confirm an almost perfectly stoichiometric oxygen content with *δ* = −0.002, see Supplementary Note 2.

### X-ray absorption spectroscopy

X-ray absorption spectroscopy measurements have been performed at the 08B beamline of the National Synchrotron Radiation Research Center (NSRRC), Taiwan.

### Magnetization measurements

The magnetic properties were studied using a Quantum Design Inc. MPMS-5XL SQUID magnetometer.

### Electrical conductivity

The measurements of electrical resistivity and specific heat were carried out using a four-probe and a standard thermal relaxation calorimetric method in a Quantum Design Inc. Physical Property Measurement System.

### Neutron measurements

Unpolarized neutron measurements have been performed at the IN8 and Thales Spectrometers at the ILL in Grenoble, France. For the elastic and inelastic measurements on the Thales spectrometer, a pyrolytic graphite (PG) (002) monochromator and analyzer as well as a velocity selector were used (*k*_f_ = 1.8 Å^−1^). On the IN8 spectrometer, very first quick inelastic scans have been made using a Si monochromator and a Flatcone analyzer array. Polarized neutron measurements have been performed at the IN12 Spectrometer at the ILL in Grenoble, France. A velocity selector was used for choosing the incident neutron wavevector *k*_i_ of 2.25 Å^−1^. A horizontally and vertically focussing PG (002) monochromator and Heusler (111) analyzer have been used. The incident neutron beam was polarized by a transmission polarizer (cavity) in the neutron guide and the measured flipping ratio amounts to 22.2.

### Data availability

The data that support the findings of this study are available from the corresponding author upon reasonable request.

## Electronic supplementary material

Supplementary Information

Description of Additional Supplementary Files

Supplementary Movie 1
