# Supplementary material for: Antiferromagnetic correlations in the metallic strongly correlated transition metal oxide LaNiO3
Source: Nat Commun. 2018 Jan 3;9:43. doi: 10.1038/s41467-017-02524-x (PMC5752676; doi:10.1038/s41467-017-02524-x)
Supplement: Supplementary file 1 — Supplementary Information [file 41467_2017_2524_MOESM1_ESM.pdf]

Supplementary Note 1

Laue X-ray diffraction

We have performed Laue diffraction measurements for different spots on one of our $LaNiO_3$ single crystals. As can be seen in Supplementary Figure 1, for each studied side (left and right side) the diffraction patterns are the same for all positions (spots) over the entire length of the crystal. This indicates, that our crystal is a single crystal. Also our single crystal has no NiO impurities visible by eyes in powder X-ray diffraction measurements (see Fig. 1 in the main text).

Supplementary Note 2

Thermogravimetric measurements

Supplementary Figure 2 shows a typical thermogravimetric (TG) measurement of one of our nickelate samples that was heated to 900°C in a flow (100ml/min.) of 20% H_2 and 80% Ar gas. The oxygen concentration amounts to ~3.005 according to the graphical analysis and ~2.998 as derived from weighing initial and final masses before and after the reduction of the sample. Both values basically indicate the absence of Ni^{2+} impurities in our $LaNiO_3$ crystals. The small difference between these values is comfortably within the typical error of TG measurements and can be attributed to the impact of the gas flow which is affecting the buoyant force that has non-negligible influence on the weighing of the sample mass during the reaction.

Supplementary Note 3

Crystal structure

Moreover, we determined the atomic positions of $LaNiO_3$ by means of single crystal X-ray diffraction. The structural parameters are listed in Supplementary Table 1 and the (obtained) crystal structure is visualized in Supplementary Figure 3.

Supplementary Note 4

Physical properties

We also re-measured the magnetic susceptibility and electrical resistivity for six different single crystals from three batches that we have grown, see Supplementary Figure 4.

If the crystal is a single crystal and not polycrystalline it shows the kink in the magnetic susceptibility and a low value of the electrical resistivity, see Supplementary Figure 4. However, if the crystal is not single crystalline, we also observe a magnetization without any kink, similar as in Supplementary Ref. [1]. Therefore, we could imagine that magnetic ordering only appears in $LaNiO_3$ single crystals where domain sizes are very large and samples are very pure.

Finally, there seems to be no field dependence (up to 9 T) of the electrical resistivity of $LaNiO_3$ - see Supplementary Figure 5. Since the single crystals are twined (and pseudocubic) also no direction dependence on the magnetic field direction can be expected.

Supplementary Note 5

Magnetic structure

Since our single crystal is fully twinned we decided to make a magnetic symmetry analysis for the high symmetry cubic (undistorted perovskite) structure with space group $Pm\bar{3}m$. The integrated intensities of the (0 0 1) and (1 1 0) nuclear peaks and (0.25 0.25 0.75), (0.75 0.75 0.25) and (0.75 0.75 0.75) magnetic peaks have been obtained from Gaussian fits to the corresponding Q scans (note, that an analyzer was used in our triple-axis measurements,) and normalized by the monitor and by the attenuation factor (plexi: 7.5) for the nuclear reflections. The scale factor was determined from the nuclear intensities and fixed for the subsequent magnetic structure refinement. The magnetic structure was determined by irreducible representation (IR) analysis for space group $Pm\bar{3}m$ and for a propagation vector (0.25 0.25 0.25). The reducible magnetic representation for the Ni ions at the $1b$ site is decomposed into two IRs as:

$$\Gamma = \Gamma_2 \oplus \Gamma_3. \qquad (1)$$

The basis vectors for the IRs are listed in Supplementary Table 2. and a comparison of observed and calculated intensities is listed in Supplementary Table 3. For the cubic symmetry the appearance of the (3/4 3/4 3/4) reflection excludes the model with longitudinal modulation of the magnetic moments (see Supplementary Figure 6(a)) which is also reflected by the large R- and weighted R values that amount to 65.0% and 69.4%.

The model with helical arrangement of the magnetic moments perpendicular to the propagation vector (see Supplementary Figure 6(b)) is consistent with our data (R- and weighted R values amount to 16.3% and 17.7% respectively) and yields an ordered moment of about 0.3 μ_B.

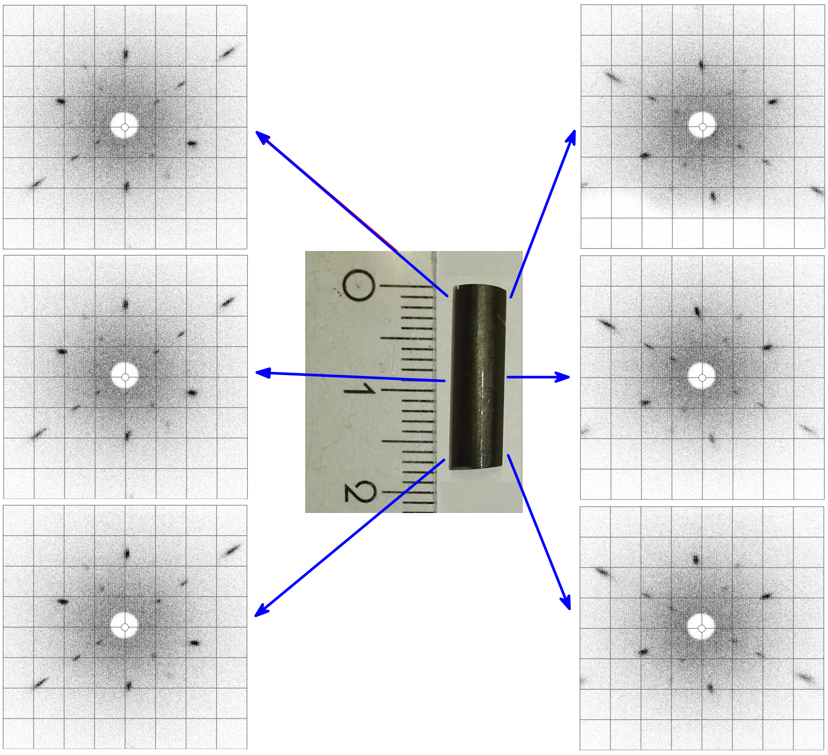

Supplementary Figure 1: Laue diffraction images A photo of one of our $LaNiO_3$ single crystals (batch 2) together with Laue diffraction images measured at different spots on the sample which are indicated by the blue arrows.

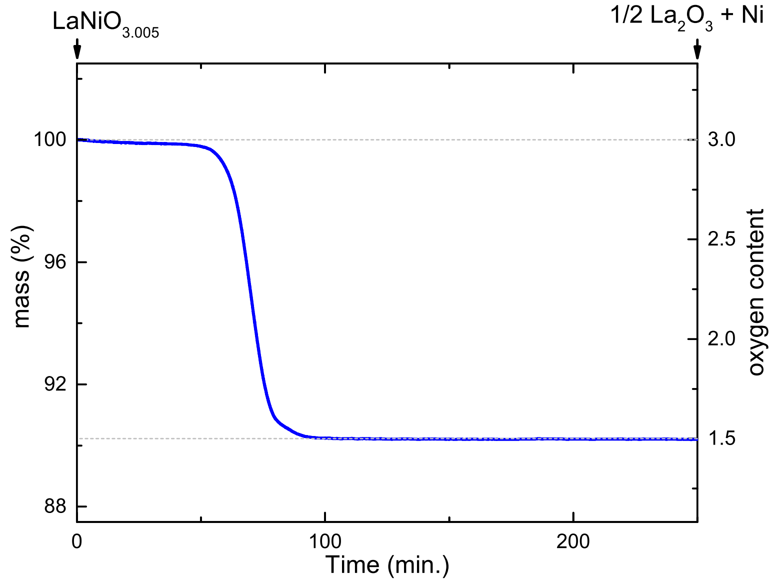

Supplementary Figure 2: TG measurements Temporal dependence of the sample mass of one of our $LaNiO_3$ crystals during the reduction process of a thermogravimetric measurement. The right scale denotes the calculated oxygen content x as a function of time assuming that the reaction $2\ LaNiO_x + 3\ H_2 \rightarrow La_2O_3 + 2\ Ni + 3\ H_2O\uparrow$ went to completion after 250 min. The grey dashed horizontal lines refer to a nominal oxygen content of $x = 3$ and 1.5 respectively.

atom	occup.	x	y	z
La1	0.995(12)	0	0	0.25
Ni1	1.000(14)	0	0	0
O1	1	0.5457(4)	0	0.25

atom	U_{11} (Å^2)	U_{22} (Å^2)	U_{33} (Å^2)	
La1	0.00523(7)	0.00523(7)	0.00803(16)	
Ni1	0.00304(13)	0.00304(13)	0.0055(3)	
O1	0.0077(5)	0.0077(9)	0.0093(11)	
**atom**	**U_{12} (Å^2)**	**U_{13} (Å^2)**	**U_{23} (Å^2)**	**U_{iso} (Å^2)**
La1	0.00261(4)	0	0	0.00616(7)
Ni1	0.00152(7)	0	0	0.00388(13)
O1	0.0038(5)	-0.0015(4)	-0.0029(7)	0.0082(6)

Supplementary Table 1: Crystal structure Refinement results of single crystal X-ray diffraction measurements of $LaNiO_3$ at room-temperature (space group $R\bar{3}c$).

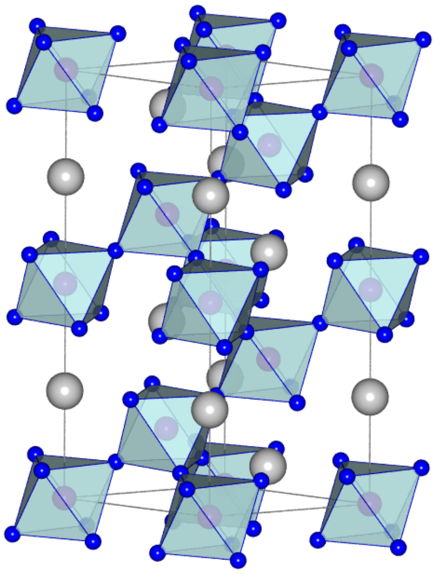

Supplementary Figure 3: Crystal structure A representatiopn of the crystal structure of $LaNiO_3$ in the hexagonal setting of space group $R\overline{3}c$ as derived from our single crystal X-ray diffraction measurement at room-temperature. Magenta/blue/white spheres: Ni, O and La ions.

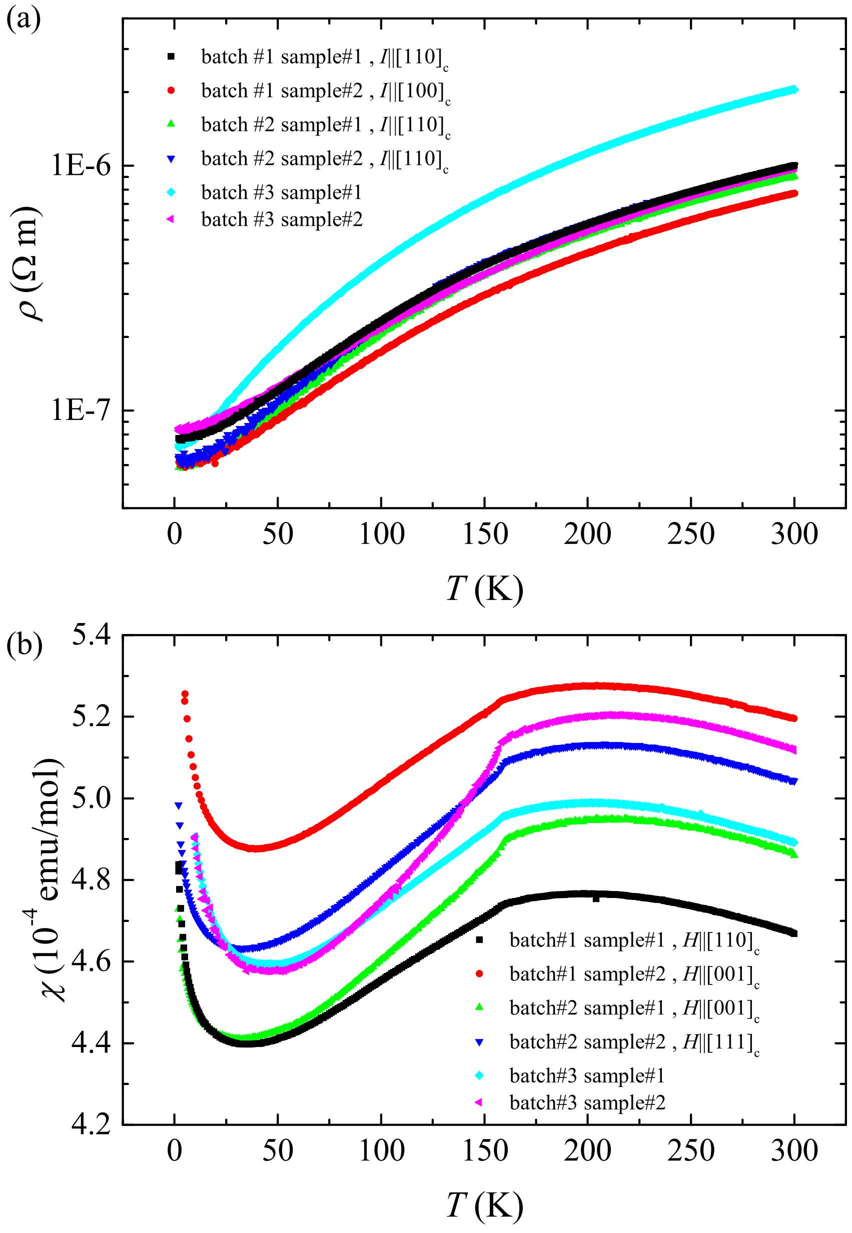

Supplementary Figure 4: Physical properties (a) Electrical resistivity and (b) magnetic susceptibility measurements which have been done for two pieces (each) of three different single crystals (batches) that we have grown.

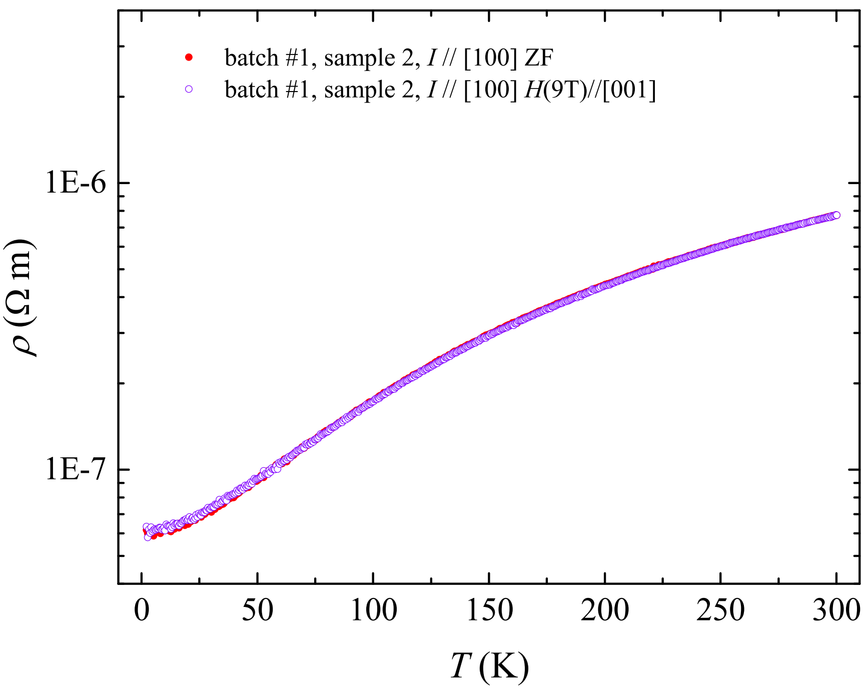

Supplementary Figure 5: Field dependence of the electrical resistivity The electrical resistivity of $LaNiO_3$ measured for different applied magnetic fields H.

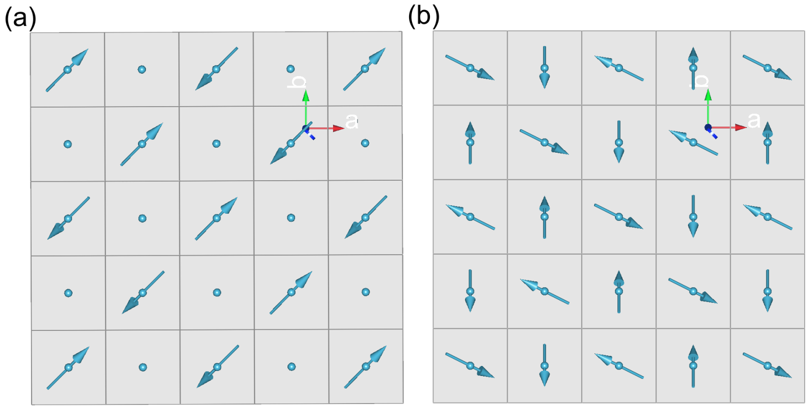

Supplementary Figure 6: Spin structure Spin configurations for models with (a) longitudinal modulation of the magnetic moments (Γ_2) and (b) helical arrangement of the magnetic moments perpendicular to the propagation vector (Γ_3).

Supplementary Table 2: Magnetic symmetry Basis vectors of the irreducible representations of the space group $Pm\bar{3}m$ for sites 1*b* with propagation vector **k** = (0.25 0.25 0.25).

Ni (1*b*)			(1/2, 1/2, 1/2)
Γ_2	ψ_1	Re	(1, 1, 1)
Γ_3	ψ_1	Re	(1, -0.5, -0.5)
		Im	(0, - $\sqrt{3}$/2, $\sqrt{3}$/2)
	ψ_2	Re	(0.5, -1, 0.5)
		Im	($\sqrt{3}$/2, 0, - $\sqrt{3}$/2)

Supplementary Table 3: Magnetic structure Comparison of the measured and calculated intensities for our two models shown in Supplementary Figure 6. I_{obs}: measured intensities, I_{cal,Γ_2}: calculated intensities for the longitudinal moment modulation, I_{cal,Γ_3}: calculated intensities for the helical moment arrangement.

magnetic peak	I_{obs}	I_{cal,Γ_2}	I_{cal,Γ_3}
(0.25 0.25 0.75)	18.3	26.5	17.4
(0.75 0.75 0.25)	27.3	13.6	21.6
(0.75 0.75 0.75)	22.2	0	26.6

Supplementary References

1. J. Zhang, H. Zheng, Y. Ren and J. F. Mitchell, High-Pressure Floating-Zone Growth of Perovskite Nickelate $LaNiO_3$ Single Crystals. *Cryst.Growth Des.* **17**, 2730-2735 (2017).
