# Supplementary material for: Antiferromagnetic correlations in the metallic strongly correlated transition metal oxide LaNiO3
Source: Nat Commun. 2018 Jan 3;9:43. doi: 10.1038/s41467-017-02524-x (PMC5752676; doi:10.1038/s41467-017-02524-x)
Supplement: Supplementary file 2 — Description of Additional Supplementary Files [file 41467_2017_2524_MOESM2_ESM.pdf]

Description of Additional Supplementary Files

File Name: Supplementary Movie 1
Description: The floating zone and the counterrotating upper and lower rods during the growth of $LaNiO_3$ within the growth chamber consisting of a hollow sapphire crystal, which is able to sustain pressures of 130-150 bar that are needed to grow single crystalline $LaNiO_3$.
